# Properties of STAT1 and IRF1 enhancers and the influence of SNPs

**DOI:** 10.1186/s12867-017-0084-1

**Published:** 2017-03-09

**Authors:** Mohamed Abou El Hassan, Katherine Huang, Manoja B. K. Eswara, Zhaodong Xu, Tao Yu, Arthur Aubry, Zuyao Ni, Izzy Livne-bar, Monika Sangwan, Mohamad Ahmad, Rod Bremner

**Affiliations:** 1 Lunenfeld Tanenbaum Research Institute, Mt Sinai Hospital, Toronto, ON Canada; 2 Clinical Chemistry Division, Provincial Laboratory Services, Queen Elizabeth Hospital, Charlottetown, PE Canada; 3 Department of Pathology, Faculty of Medicine, Dalhousie University, Halifax, NS Canada; 4 Department of Lab Medicine and Pathobiology, University of Toronto, Toronto, ON Canada; 5 Department of Ophthalmology and Vision Science, University of Toronto, Toronto, ON Canada; 6 Donnelly Centre, University of Toronto, Toronto, ON Canada

## Abstract

**Background:**

STAT1 and IRF1 collaborate to induce interferon-γ (IFNγ) stimulated genes (ISGs), but the extent to which they act alone or together is unclear. The effect of single nucleotide polymorphisms (SNPs) on in vivo binding is also largely unknown.

**Results:**

We show that IRF1 binds at proximal or distant ISG sites twice as often as STAT1, increasing to sixfold at the MHC class I locus. STAT1 almost always bound with IRF1, while most IRF1 binding events were isolated. Dual binding sites at remote or proximal enhancers distinguished ISGs that were responsive to IFNγ versus cell-specific resistant ISGs, which showed fewer and mainly single binding events. Surprisingly, inducibility in one cell type predicted ISG-responsiveness in other cells. Several dbSNPs overlapped with STAT1 and IRF1 binding motifs, and we developed methodology to rapidly assess their effects. We show that in silico prediction of SNP effects accurately reflects altered binding both in vitro and in vivo.

**Conclusions:**

These data reveal broad cooperation between STAT1 and IRF1, explain cell type specific differences in ISG-responsiveness, and identify genetic variants that may participate in the pathogenesis of immune disorders.

**Electronic supplementary material:**

The online version of this article (doi:10.1186/s12867-017-0084-1) contains supplementary material, which is available to authorized users.

## Background

IFNγ is a pleiotropic cytokine that plays essential roles in antiviral and anticancer immune responses (reviewed in [1, 2]). IFNγ binds to its receptor complex and activates receptor-associated JAK kinases, which phosphorylate a substantial fraction of cytoplasmic signal transducer and activator of transcription 1 (STAT1). Phosphorylated STAT1 forms homodimers that translocate to the nucleus and bind IFNγ activation sites (GAS). STAT1 recruits histone acetyltransferases (HATs) and other transcriptional co-activators to acetylate chromatin and facilitate transcription. Genomic studies showed that STAT1 binds at promoter proximal and distal sites, suggesting a role in remote gene regulation [3–6]. Indeed, IFNγ induces long range interactions between STAT1-bound enhancers and target promoters [7–9].

Interferon regulatory factor 1 (*IRF1*) is a primary target gene of STAT1. Like STAT1, IRF1 also acts as a transcription factor (TF), binding to IRF-E motifs and interferon-stimulated response elements (ISRE) [10, 11]. Access of both STAT1 and IRF1 to target enhancers requires the SWI/SNF chromatin remodeling complex to counter PRC2, which uses the histone methyl transferase EZH2 to deposit H3K27me3 and block the induction of many other cytokine and cytokine responsive loci [7, 12, 13]. IRF1 functions at the transcription initiation level by facilitating RNA Pol II recruitment to ISGs promoters [14, 15]. IRF1 also binds to remote enhancers of the *CIITA* locus that loop together to form a 3D interconnected hub with the promoter [7]. Indeed, ChIP-chip and ChIP-seq studies show that IRF1 binds many remote enhancers [6, 16–18], and analysis of 128 transcription factors in K562 cells revealed that STAT1-IRF1 co-binding is a recurring pattern in IFNγ treated cells [19]. Notably, STAT1 is essential but not sufficient for gene induction [11], and both STAT1 and IRF1 are required for the IFNγ-induced expression of *CIITA*, *GBP1*, and gp19 [14, 15, 20]. In addition, STAT1 complexes with IRF1 at the *LMP2* promoter and maintains its constitutive expression [21].

Here, we studied the extent of STAT1 and IRF1 cooperation in HeLa cells within ISG-rich chromosomal segments encompassing ~10% of all known ISGs. Most of these loci responded to IFNγ in HeLa cells, leaving ~20% resistant ISGs. IRF1 binding sites outnumbered STAT1 sites 2 to 1. A large fraction of STAT1/IRF1 binding occurred at remote sites and looping studies confirmed the functional role of putative enhancers at the *SOCS1* locus. Most STAT1 binding occurred at or near to IRF1 sites (dual binding), but IRF1 often bound isolated from STAT1. Dual STAT1 and IRF1 but not isolated IRF1 or STAT1 binding was linked to ISG responsiveness. Finally, several variants affecting STAT1/IRF1 motifs induce or impair binding.

## Results

### Diverse gene responses to IFNγ

**Fig. 1:**
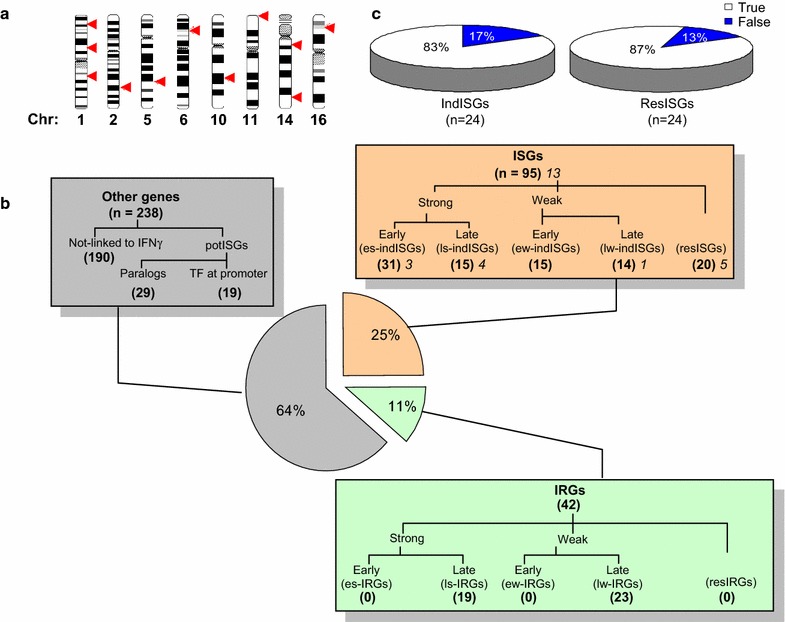
IFNγ target subclasses. **a** Human chromosome ideograms, drawn using NCBI Map Viewer, show locations of the 11 studied chromosomal segments (*red arrows*). **b** The 382 genes within these regions were classified as “IFNγ stimulated genes” (ISGs), “IFNγ-repressed genes” (IRGs) or “other genes” according to our expression array data and on prior studies (see “Methods” and “Results” sections for details). Known ISGs (in this and other studies) were either induced (indISGs) or resistant (resISGs) in HeLa cells. Genes were further subcategorized according to their robustness (strong/weak) and timing (early/late) of induction/repression (e.g. early strong induced ISGs: es-indISGs). Other genes were termed “not-linked to IFNγ”, or “potential ISGs” (potISGs) if they were either ISG paralogs or exhibited IFNγ-induced TF binding at their promoter. Italics indicate the number of CIITA targets in each class. **c** To validate array data, mRNA was isolated from HeLa cells left untreated or exposed to IFNγ for 6 h. RT-PCR was performed on 24 induced (IndISGs) or resistant (ResISGs) ISGs, selected based on array data 6 h after IFNγ treatment. *Pie diagram* shows the percentage of validated genes

To define patterns of TF binding around ISGs, we employed tiling arrays to focus on 16 Mb distributed across 11 distinct chromosomal segments with a high density of ISGs (Fig. 1a, Additional file 1: Table S1). Nine segments were 1 Mb genomic regions on six chromosomes centered on specific IFNγ target genes (e.g. 1 Mb around *IRF1* etc.). Two others included a 2 Mb segment centered on *CIITA*, and a 5 Mb segment covering the complete classical 3.6 Mb MHC locus and an additional 1.4 Mb 5′ region including much of the so-called extended MHC class I region (Fig. 1a; Additional file 1: Table S1). Within these regions 25% (95/375) of the genes are known ISGs, ~5× more than the genome-wide ISG frequency (1167/24996 ISGs) and ~15-fold above the average ISGs density per Mb. The total number of Refseq genes and UCSC Known Genes in the 16 Mb regions is 394 (Additional file 1: Table S1). Of these, 95% (375) were represented on the Illumina-12 Human WG-6v3 array used to assess gene expression (see below). The frequency of Refseq genes across the genome is ~6/Mb, but most of the 11 chromosomal segments in our study were gene dense (average 24/Mb), especially at the MHC (35/Mb), PSME (39/Mb) and IFITM clusters (45/Mb) (Additional file 1: Table S1). There are also 126 pseudogenes across the 16 Mb, with most (93) located at the MHC cluster (Additional file 1: Table S1). Pseudogenes are not represented on the Illumina genome wide array we used to study expression.

Signaling pathway target loci show cell-specific responsiveness, but the exact TF binding patterns that distinguish induction versus resistance in a specific cell type are unclear. Thus, we compared the pattern of STAT1 and IRF1 binding at different gene types. For this we compiled a database of ~ all known ISGs using our own and prior transcriptome data (Additional file 1: Table S2). As summarized in Fig. 1b, ISGs fell into 8 classes depending on whether IFNγ caused induction, no effect (resistant ISGs in HeLa cells), or repression, and whether induction/repression were early (detected at 6 h) or late (24 or 48 h), and strong (differential score ≥13, and ≥twofold change) or weak (differential score ≥13, <twofold). The microarray expression data was validated using reverse transcription and quantitative PCR (RT-qPCR), which confirmed 83% (20/24) of indISGs and 87% (21/24) of resISGs (Fig. 1c). Of all 95 known ISGs on the array, 31 (33%) genes were es-indISGs, 15 (16%) were ls-indISGs, 29 (31%) were ew-indISGs or lw-indISGs, and 20 were resISGs (Fig. 1b). Es-indISGs were distributed at an average density of 1.9/Mb within the studied regions (Additional file 1: Table S1). The highest density was observed at the IFIT and GBP clusters with an average of 4.0 es-indISGs/Mb.

No genes were repressed (IFNγ repressed genes, IRGs) at the early 6 h time point, while 19 and 23 were **s**trongly or **w**eakly repressed at later times, respectively (ls-IRGs and lw-IRGs; Fig. 1b), suggesting indirect regulation of IRGs (perhaps through activation of a repressor). The remaining genes that were not IFNγ-responsive either in this or any prior study were termed “Other Genes”. In summary, known ISGs fall into induced and resistant subclasses in HeLa cells, providing a useful system to define STAT1 and IRF1 binding patterns linked to responsiveness.

### Validation of STAT1 and IRF1 ChIP-chip analyses

**Fig. 2:**
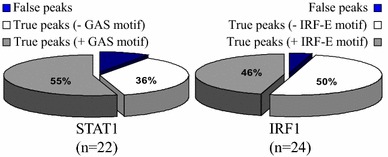
STAT1 and IRF1 binding patterns. Arbitrarily selected ChIP-chip STAT1 (n = 22), IRF1 (n = 24) sites were re-examined by ChIP-qPCR on chromatin from HeLa cells with no or 6 h of IFNγ treatment. 91% of peaks were validated in both cases. The frequency of consensus motifs identified by JASPER within STAT1 (GAS) or IRF1 (IRF-E) peaks is indicated

ChIP-chip was used to locate STAT1 and IRF1 sites at promoter proximal and distal sites of the genes of each category. STAT1 and IRF1 ChIPs were performed on chromatin from HeLa cells that were either untreated or exposed to IFNγ for 6 h. Hybridization intensities were normalized to internal standards and values from quadruplicate spots were averaged. Significantly different intensities between ChIP DNA and input DNA samples in three biological replicates (p < 0.0001) were determined using the Wilcoxon rank sum test. Peaks representing the significantly enriched DNA regions (p < 0.0001) where the ratio of ChIP to input DNA was ≥ 1.5-fold were visualized in the UCSC browser and plotted on a log2 scale. Only 2 STAT1 and 28 IRF1 peaks were identified in untreated cells, rising to 92 and 196 post-IFNγ treatment, respectively. Browser views are shown in Additional file 2: Figure S1 and can be visualized at http://research.lunenfeld.ca/IFNy. ChIP-qPCR validated 91% (20/22) and 96% (23/24) of STAT1 and IRF1 ChIP-chip peaks, respectively (Fig. 2). We compared STAT1 binding at 6 h (this study) with IFNγ-induced STAT1 binding after 30 min [22], also assessed in HeLa cells. In the 16 Mb of DNA assessed here, the latter study detected 26 STAT1 sites, of which 21 overlapped with the 92 STAT1 sites we detected.

### Basal TF binding

Unphosphorylated STAT1 has roles in regulating ISGs days after IFN treatment [23, 24], but its role in untreated cells is less clear, although STAT1 nuclear cytoplasmic shuttling occurs even in untreated cells [25–27]. Basal STAT1 binding is linked to the nuclear localization of unphosphorylated STAT1 and contributes to the constitutive expression of some targets [21, 28]. IRF1 is also expressed to low levels in unstimulated HeLa cells [7] and it cooperates with STAT1 to maintain low basal expression levels of LMP2 [21]. In addition, there is also some STAT1 phosphorylation (below detectable levels) in untreated cells that contributes to basal activity, as shown elegantly by knockin studies in mice [29]. We detected 2 STAT1 and 28 IRF1 binding sites in untreated cells, accounting for 2.2 and 14.3% of induced sites, respectively. Our data accords with another ChIP-chip analysis of STAT1 binding which reported that 6.5% of IFNγ-induced STAT1 sites in HeLa cells treated for 30 min with IFNγ (as opposed to 6 h in our case) are occupied in uninduced cells [22].

Further analysis suggests that basal TF binding detected here is physiologically relevant. Of the genes with basal STAT1 or IRF1 binding, we assessed 21 by microarray and/or RT-PCR and all were expressed in untreated cells (Additional file 1: Table S3). In contrast, of 26 randomly selected ISGs that lacked basal TF binding, only 13 were basally expressed. Indeed, constitutive expression of PSMB9 and TAP2 requires constitutive IRF1 binding [21, 30]. In addition, several loci with basal TF binding are in paralogous gene clusters suggesting conservation of high affinity binding sites during gene duplication (e.g. PSMB8 and PSMB9, GBP2 and GBP3, and IFIT1, IFIT2 and IFIT3). A high fraction (82%) of the 28 IRF1 basally occupied sites possessed IRF1 binding motifs. Thus, our data supports the notion that basal binding of STAT1 and IRF1 is physiologically relevant.

### Remote IFNγ activated enhancers are common at ISGs

**Fig. 3:**
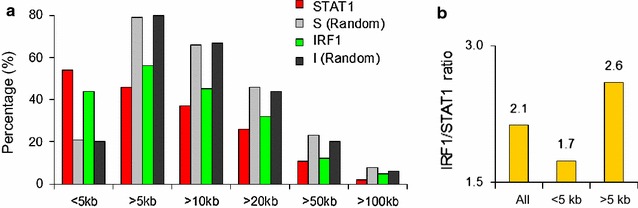
Proximal *vs.* distal STAT1 and IRF1 binding. **a** The percentage of IFNγ-induced STAT1 or IRF1 binding sites (*top*) or randomly generated controls at proximal (≤5 kb) or distal (>5 kb) sites relative to the TSS of Known genes. **b** IRF1 to STAT1 ratio at proximal and remote sites

In IFNγ treated cells, 54% (50/92) of STAT1 and 44% (87/196) of IRF1 peaks were within 5 kb of the transcription start site (TSS) of all 394 Known Genes on the array (Fig. 3a; Additional file 1: Table S4). Adding other databases, including predicted genes, raised the fraction to 64% for STAT1 and 57% for IRF1 (Additional file 2: Figure S2A). Of equal numbers of randomly generated sites, the proportion at <5 kb from gene starts was much lower (Fig. 3a; Additional file 2: Figure S2A). Thus STAT1 binding is slightly skewed to promoter proximal sites, while IRF1 binding is slightly biased toward remote sites (Fig. 3b).

**Fig. 4:**
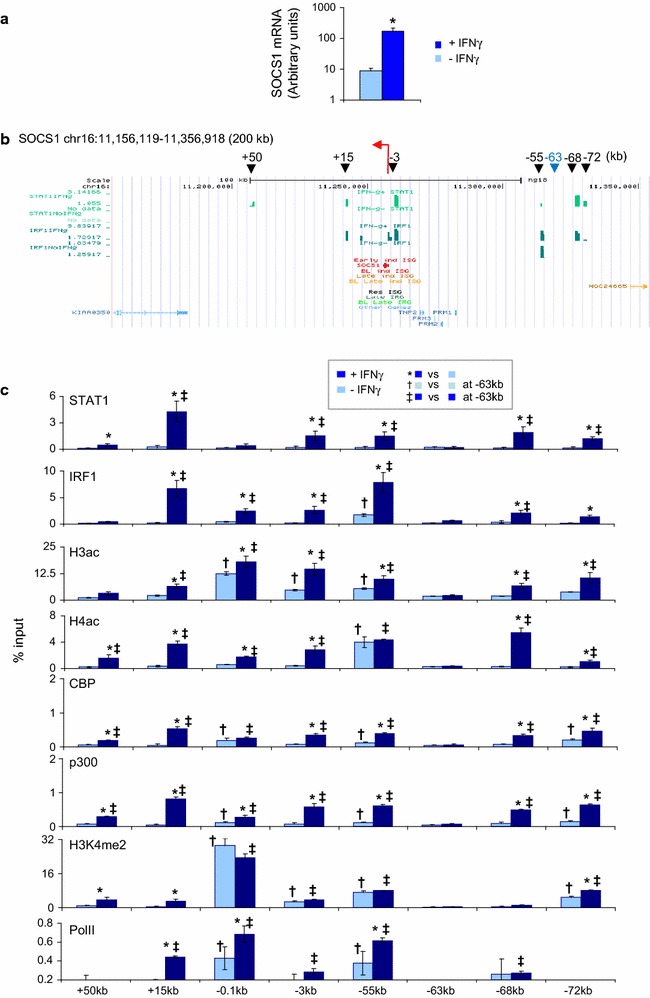
STAT1 and IRF1 binding at *SOCS1*. **a** RT-qPCR for SOCS1 mRNA in HeLa cells treated with IFNγ for 0 or 6 h. Data are in arbitrary units relative to β-actin levels (log scale). **b** ChIP-chip maps of STAT1 and IRF1 binding across the *SOCS1* locus treated as in **a**. *Black arrowheads* indicate TF binding sites of interest, with distances from the TSS (*red arrow*) in kb. The TF-free −63 kb site (*blue*) is used as a negative control in **c**. **c** ChIP-qPCR analysis of the basal and IFNγ-induced histone modifications or recruitment of the indicated factors. Marks (*^, †, ‡^) show significant differences (p < 0.05, ANOVA followed by Fisher test) in the indicated comparisons (mean ± SD, n = 3)

Prior analysis of chromatin modification and looping at *CIITA*, and partial looping analysis at *1* locus support the idea that remote sites are functionally important [7, 8]. To further test this notion we performed additional assessment of *SOCS1*, a key negative regulator of IFNγ signaling that is responsive to IFNs and other immune signaling pathways [31, 32]. *SOCS1* responds to IFNγ in HeLa cells (Fig. 4a). ChIP-chip data exposed 6 IFNγ-induced STAT1 and/or IRF1 peaks ± 100 kb of the *SOCS1* TSS (Fig. 4b). ChIP-qPCR analysis verified binding at the *SOCS1* promoter (*pSOCS1;* −*0.1* *kb*), and at +50, +15, −3, −55, −68 and −72 kb (Fig. 4c). A negative region at −63 kb was also validated. In favor of functional relevance of proximal and remote sites, we detected constitutive histone H3 acetylation (H3ac) and/or H4ac at pSOCS1 (−0.1 kb), −3 and −55 kb (Fig. 4c), and IFNγ induced acetylation at pSOCS1 and the 6 remote sites but not at the irrelevant −63 kb site (Fig. 4c). These constitutive and inducible events paralleled recruitment of the HATs CBP and/or p300 (Fig. 4c). H3K4me2 also marks enhancers [33], and constitutive H3K4me2 was detected at *pSOCS1*, −3 kb, −55 kb, matching constitute histone acetylation, and also at the −72 kb enhancer (Fig. 4c), which contacts the promoter (see below). IFNγ treatment did not further increase methylation at these sites, but did induce H3K4me2 at +50 kb, +15 kb and −72 kb (Fig. 4c). Finally, we detected constitutive Pol II recruitment at *pSOCS1* and −55 kb but not at the other TF binding or negative control sites (Fig. 4c). After IFNγ treatment, Pol II recruitment increased at *pSOCS1*, +15 kb and −55 kb (Fig. 4c). Association with the +15 kb element may reflect IFNγ-induced promoter looping (see below).

**Fig. 5:**
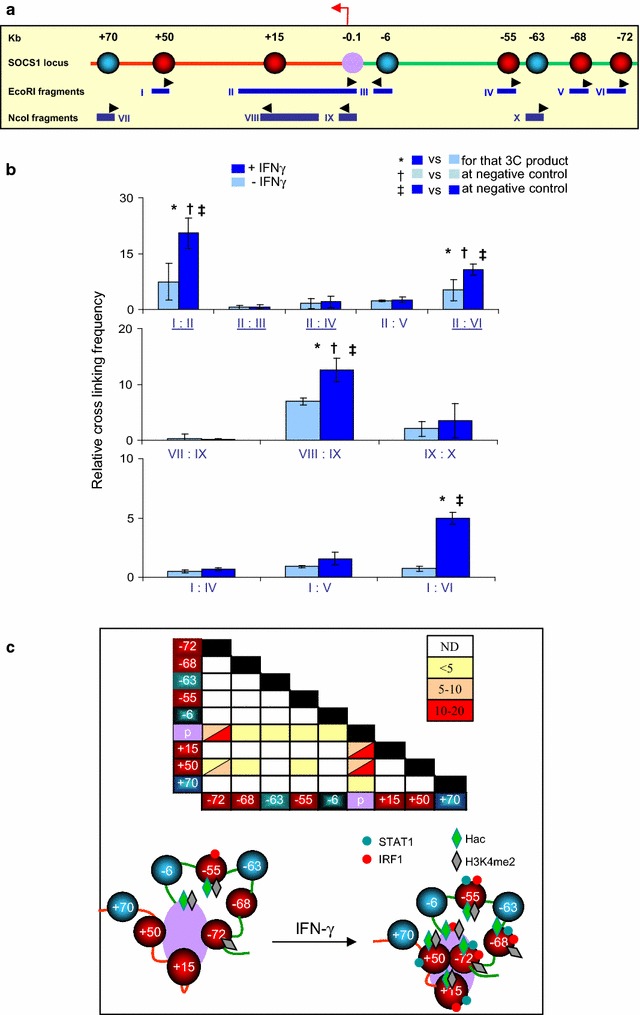
Basal and IFNγ-induced looping at *SOCS1*. **a** A schematic view of the *SOCS1* locus. *Circles* indicate the *SOCS1* promoter (*purple*), putative remote enhancers (*red*), and negative control sites (*blue*), with distances from the TSS (*red arrow*) indicated above in kb, while fragments used in 3C assays with primers (*black arrowheads*) are shown below. **b** Cross linking frequencies between the promoter and remote sites across the *SOCS1* locus. Quantitative 3C was performed with chromatin from HeLa cells left untreated or exposed to IFNγ for 6 h. Bar graphs show the crosslinking frequency of a selected number of interactions. *Underlined* interactions were published previously [8]. Marked interactions (*^, †, ‡^) are significantly different at the indicated comparisons (p < 0.05, ANOVA followed by Fisher test, mean ± SD, n = 3). **c** Summary of looping events. Interacting sites and DNA strands are colored as in **a**. STAT1/IRF1 (*green*/*red dots*) and Hac/H3K4me2 (*green*/*gray diamonds*; data from Fig. 6c) are also depicted

Chromosome conformation capture (3C) revealed both constitutive and IFNγ-induced contacts between the promoter and remote STAT1 and/or IRF1 sites at *CIITA* [7]. To examine looping at *SOCS1*, we studied six EcoRI (I–VI) or four NcoI (VII–X) fragments (Fig. 5a). Of a total of 22 possible interactions, we studied 4 previously ([8]; underlined in Fig. 5b) and assessed an additional 7 in this study. As expected, no interaction was observed between fragments containing the promoter and irrelevant sites at −63, −6, or +70 kb, either before or after cytokine exposure. However, between suspected functional elements, we detected a total of 3 loops of all 11 putative interactions in the basal state, and each of these loops was enhanced after IFNγ treatment and was accompanied by a new interaction between the +50 and −72 kb enhancers that lie 122 kb apart (Fig. 5b, c). Our data suggest that the SOCS1 locus is basally present in a mega looping complex that becomes more compact and involves more inter-element interactions after IFNγ treatment. Together, the ChIP and 3C data show that STAT1 and IRF1 binding is linked to extensive chromatin modifications and looping.

### Unusual IRF1 distribution at MHC loci

As noted earlier, IRF1 exceeded STAT1 sites by ~twofold, but this varied at some regions, most notably at the MHC class I locus where the ratio was 3.5:1 (56 IRF1:16 STAT1 sites; Additional file 1: Table S4). The ratio was particularly skewed at the extended (6:1) versus classic (2.9:1) MHC class I region. 26 of all MHC class I IRF1 sites were within 5 kb of Known Gene starts and 17 within 5 kb of pseudogenes (Additional file 1: Tables S4, S5), giving a total of 77% (43/56) promoter proximal sites, which is higher than the 44% at all loci (Fig. 3a; Additional file 1: Table S4). However, whereas 66% (25/38) of IRF1 sites were promoter-proximal in the classical MHC class I region, this dropped to only 6% (1/18) at the extended MHC class I region, and was low even after including pseudogenes (4/18; Additional file 1: Tables S4, S5), leaving an unusually high fraction of remote IRF1 sites (78%). Thus, IRF1 seems to play a broader role than STAT1 at the MHC class I cluster, primarily at proximal elements in the classic region, but at remote elements in the extended region.

STAT1 and IRF1 induce *CIITA*, the master regulator of MHC class II expression (reviewed in [34]). The number of STAT1 and IRF1 sites was typically very low in the MHC class II region (Additional file 1: Table S4). Out of 13 MHC class II genes, 5 (DRB5, DQB1, DQB2, DQA2, and DOA) were resistant to IFNγ in HeLa cells, 5 (DOB, DRB1, DQA1, DPA1, DPB1) responded only after 24 h, a time of maximum production of CIITA [35], and 3 (DRA, DMB and DMA) were es-indISGS. With the exception of DOB, none of the resistant or late-induced genes exhibited STAT1 or IRF1 promoter binding. However, of the 3 es-indISGs, two had promoter proximal IRF1 binding while DMA had IRF1 binding fairly near (~8 kb) its promoter. Thus IRF1 may cooperate with CIITA at a subset of MHC class II promoters. Others reported CIITA-independent induction of MHC class II genes [36–39], which may, therefore, involve IRF1.

### STAT1 and IRF1 binding is enriched at robustly induced ISGs

**Fig. 6:**
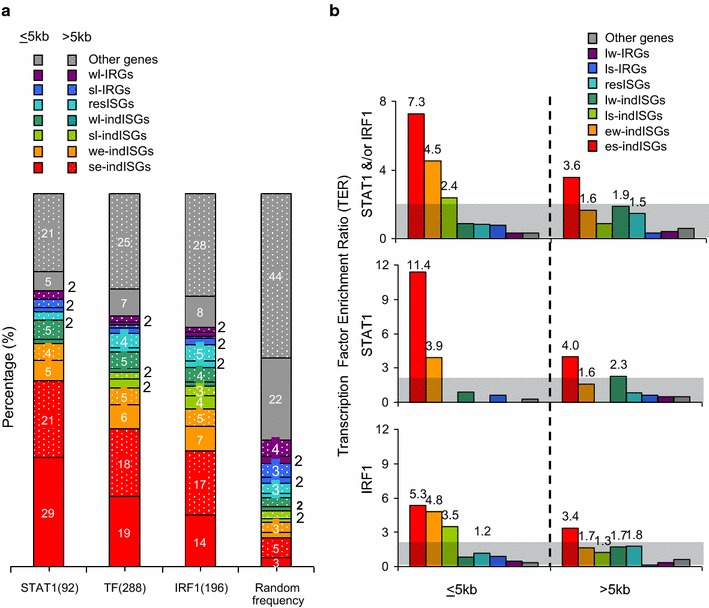
Enrichment of STAT/IRF1 at strong early induced ISGs. **a** Histogram shows the percent of STAT1, IRF1, or an equal number of randomly generated sites at proximal (<5 kb) or distal (>5 kb) sites of ISGs (excluding CIITA target genes), IRGs, and other genes. The number of sites in each category is indicated below each bar. “Random frequency”: distribution of 288 randomly generated sites. **b** Distribution of TFs normalized to that of random controls. Unshaded region indicates TER > twofold

As discussed, ISGs fell into 8 classes depending on whether IFNγ caused induction, no effect (resistant ISGs in HeLa cells), or repression, and whether induction/repression were early or late, and strong or weak (Fig. 1b). We plotted the distribution of STAT1 and IRF1 binding sites relative to all 8 gene classes. Binding sites were assigned to the nearest gene class, designated as proximal or distal when ≤5 or >5 kb from the TSS, respectively, and were compared to 288 randomly chosen sites equaling number of STAT1 + IRF1 sites (Fig. 6a). We also calculated the TF enrichment ratio (TER) in which the % distribution of TFs at proximal and distal locations was normalized to the % distribution of random sites (Fig. 6b). A binding frequency twice that of random sites (TER = 2) was assigned as an arbitrary minimum threshold.

STAT1 and IRF1 binding sites were most highly associated with robustly induced IFNγ targets (es-indISGs; Fig. 6). This applied when STAT1 or IRF1 were considered together, separately, and at proximal or remote locations (Fig. 6b). Consistent with this finding, weakly induced genes (ew-indISGs) had fewer binding events and lower TERs (Fig. 6). Of 236 Other genes (never classified as an ISG in any study), a total of only 17 had 9 STAT1 and 16 IRF1 proximal peaks, mostly (13/17) located at the MHC and RT-qPCR confirmed no induction at 10/10 of these genes (Additional file 1: Table S6). IFNγ enhancers loop over large distances at *CIITA* [7] and *SOCS1* (Fig. 5), so proximal and distal enhancers nearest to Other Genes may target neighboring ISGs. In summary, the data indicate a clear bias of STAT1 and IRF1 binding at rapidly and robustly induced ISGs, but not other gene classes.

### Isolated or dual STAT1 and IRF1 recruitment is directed by binding motifs

**Fig. 7:**
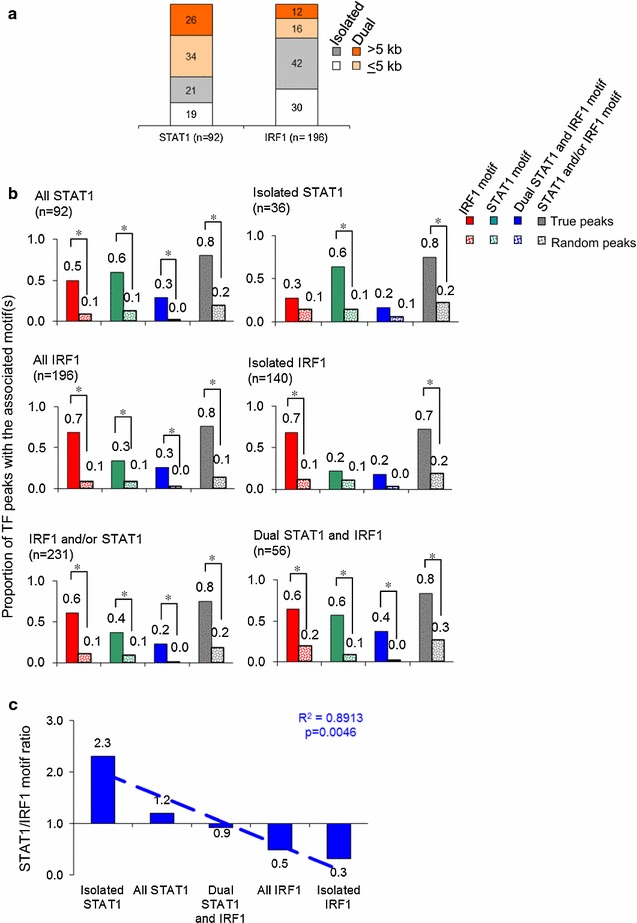
STAT1 and IRF1 binding correlates with binding motifs. **a** Percent distribution of isolated STAT1 or IRF1 or dual STAT1 + IRF1 binding at proximal (≤5 kb) or remote (>5 kb) sites of Known gene promoters. **b** TF binding sites were classified into 6 subclasses, then mapped motifs using CisGenome’s “Known Motif Mapping” program (see “Methods” section for details). Sets of equal numbers of randomly generated “peaks” were used to define the background occurrence of STAT1 and IRF1 motifs. *Asterisk* indicates significant difference between true and random peaks (p < 0.00005, two-sided probability test in R). **c** Ratio of STAT1/IRF1 motifs at different categories of peaks

Next we compared the fraction of isolated or dual STAT1/IRF1 binding events. Of a total of 230 discrete TF binding regions, 16% (36/230) exhibited STAT1 binding alone (isolated STAT1), of which almost half (17/36) were proximal; 61% (140/230) exhibited only IRF1 binding (isolated IRF1), of which 42% (59/140) were proximal; and 23% (54/230) showed overlap (dual STAT1/IRF1), of which slightly more than half (31/54) were proximal (Additional file 1: Table S4). Randomly generated sites showed negligible overlap (Additional file 2: Figure S2B), but dual STAT1/IRF1 binding represented more than half (54/92; 59%) of all STAT1 sites and about a quarter (54/196; 28%) of IRF1 peaks (Fig. 7a). Fewer overlapping IRF1 peaks reflect their twofold excess relative to STAT1 peaks. Thus, STAT1 preferentially binds with IRF1 at IFNγ enhancers, whereas most IRF1 sites are not co-localized with STAT1.

JASPER analysis of IRF1 and STAT1 peak regions revealed that the cognate binding motif was observed at a statistically significant level relative to equal numbers of random peaks (Fig. 7b). 60% of isolated STAT1 peaks had a STAT1 motif, and only 30% had an IRF1 motif, while 70% of isolated IRF1 peaks possessed an IRF1 motif, but only 20% had a STAT1 motif. A strong correlation existed between STAT1/IRF1 binding and the presence of the corresponding motifs (Fig. 7c). Indeed 40% of dual STAT1/IRF1 sites had both binding motifs, whereas there were none at equal numbers of randomly generated sites (Fig. 7b). Dual sites which have only a STAT1 or IRF1 binding motif may reflect protein–protein interaction or DNA looping as seen at the *SOCS1* and *CIITA* loci (Fig. 4c) [7, 8]. In summary, DNA sequence directs isolated or dual STAT1/IRF1 binding in IFNγ treated cells.

### Dual STAT1 and IRF1 targeted enhancers distinguish responsive from resistant ISGs

**Fig. 8:**
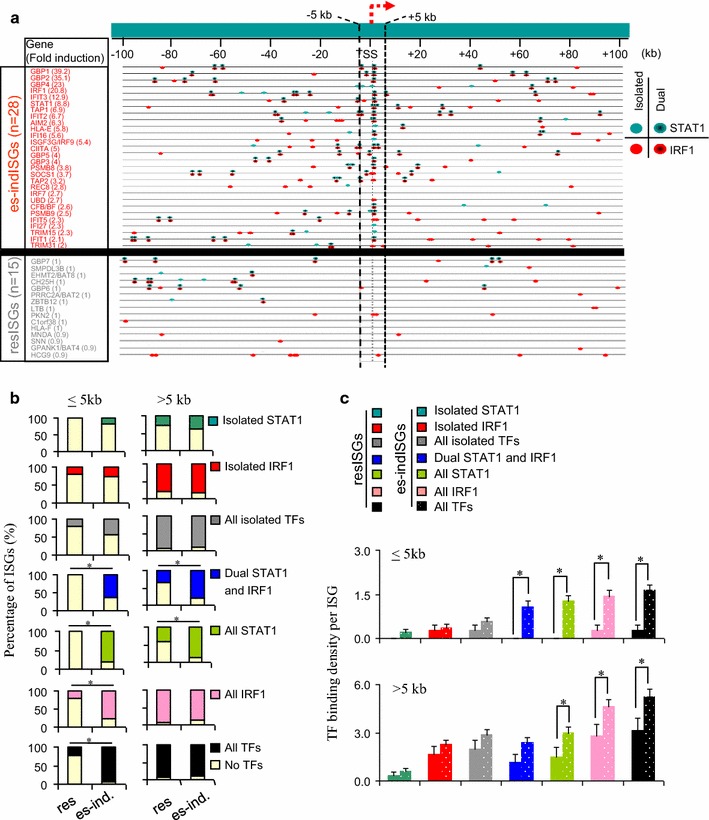
STAT/IRF1 recruitment at es-indISGs vs res-ISGs. **a** Map of isolated and dual STAT1 and IRF1 binding ± 100 kb of the TSS of es-indISGs (*top*) and resISGs (*bottom*), after removing CIITA targets, in HeLa cells treated for 6 h with IFNγ. *Red arrow* indicated TSS and direction of transcription. Genes are ranked according to fold induction, indicated in *brackets*. **b** Histograms show the percentage of es-indISGs or resISGs with proximal (≤5 kb) or distal (>5 kb) binding of STAT1 and/or IRF1. *Asterisk* p < 0.05, Fisher exact test. **c** Average number of TF binding events at proximal and remote sites at resISGs or es-indISGs. Error bar: SEM; *Asterisk* p < 0.05, ANOVA followed by Fisher test

Comparing inducible ISGs in our array study with ~all known ISGs in a large database (Additional file 1: Table S2) revealed resistant ISGs (res-ISGs) in HeLa cells (Fig. 1b). There were far fewer STAT1/IRF1 binding events at res-ISGs vs ind-ISGs, and the TER (ratio of actual TF binding to random sites) at res-ISGs was low, and similar to that at other genes (Fig. 6). There was near ubiquitous association of both STAT1 and IRF1 at es-indISG promoters, but they were virtually absent at res-ISG promoters (Fig. 6). To quantify the types of TF binding events (isolated, dual, etc.), we plotted the frequency of genes with at least one binding event within or beyond 5 kb (Fig. 8b), and the density of each type of binding event per gene (Fig. 8c). Isolated TF binding did not discriminate the two gene classes, whereas there was significantly more dual STAT1 and IRF1 binding at esISGs, at both proximal and distal sites (Fig. 8b, c). Thus, cooperation between STAT1 and IRF1 plays a central role in mediating IFNγ responsiveness.

### Degree of TF binding and responsiveness in HeLa predicts ISG responsiveness in other cell types

**Fig. 9:**
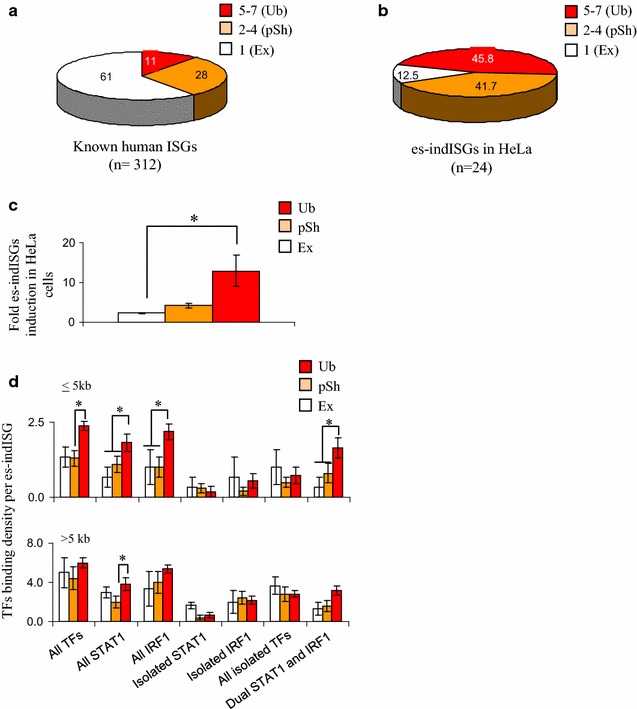
Link between ISG responsiveness in HeLa cells, responsiveness in other cell types, and STAT1/IRF1 binding. **a** Percentage of ubiquitous (Ub), partially shared (pSh), or exclusive (Ex) human ISGs based on their responsiveness to IFNγ in the indicated number of cell lines. **b** Percentage of HeLa es-indISGs which respond in only HeLa or in more cell types (as in **a**). For full lists of ISGs and es-indISGs see Additional file 1: Tables S7, S8. **c** Level to which Ub, pSh or Ex es-indISGs are induced in HeLa cells. **d** Average number of TF binding at promoter (≤5 kb) and remote (>5 kb) sites of the indicated types of es-indISGs. Error bar indicates SEM; *Asterisk* p < 0.05, ANOVA followed by Fisher test

Many studies have analyzed IFN-gene responsiveness, but a comprehensive analysis of which ISGs show broad or cell-type specific expression and, more importantly, the mechanism underlying such variability, has not been attempted. To assess variability in ISG induction, we compiled expression data on ISGs from 7 different human cell lines or primary cells, including 5 listed in Additional file 1: Table S2, plus HeLa cells (this work) and BRG1-reconstituted SW13 cells [13]. Across all 7 cell lines there were a total of 312 ISGs, the majority (61%) were exclusively induced in only one cell type, 28% were induced in 2–4 cell types, and 11% were induced in most (5–7) cell types (Fig. 9a; Additional file 1: Table S8). Only 9 genes were induced in every context and these included STAT1 and IRF1, in line with their apical role in IFNγ signaling.

We assessed the relationship between broad responsiveness, degree of induction, and STAT1/IRF1 binding. HeLa ChIP-chip data provided STAT1 and IRF1 binding information for 24 es-indISGs present in all 7 expression array datasets. Of these, 3/24 were induced exclusively in HeLa, 10/24 were induced in 2–4 lines and 11/24 were induced in 5–7 lines (Fig. 9b). Of note, genes induced exclusively in HeLa were up-regulated to a much lower extent than ubiquitously IFNγ-responsive targets (Fig. 9c). Greater induction of ubiquitously responsive loci was paralleled by a higher density of TF binding at promoter proximal sites (Fig. 9d). Thus, the level of induction is linked to the degree of STAT1 and IRF1 recruitment, and there is an unexpected link between the strength of ISG induction in one context (HeLa in this case) and competency to respond to IFNγ in other contexts.

### SNPs modulate STAT1 and IRF1 binding in vitro

Defects in IFNγ signaling are linked to a wide range of disorders [40–44]. Several studies focused on the association between genetic variants and the risk of IFNγ related disorders, but at gene promoters or coding regions of ISGs rather than IFNγ responsive enhancers. Within the 16 Mb of DNA around ISGs studied here, there are a total of 7.1 × 10^5^ dbSNPs [hg19; SNPs (141)]. Of these, 6648 dbSNPs lay within the 230 STAT1/IRF1 peaks. Only 7 of these 6648 dbSNPs were listed on the GWAS database. GWAS SNPs do not define all disease associated SNPs (DA-SNPs) because GWAS genotyping arrays provide low genomic coverage [45] and therefore the 6648 dbSNPs may encompass other DA-SNPs not mapped yet. None of the 7 DA-SNPs (GWAS database) overlapped with a STAT1/IRF1 motif, but 80 of the 6648 dbSNPs overlapped with 27 STAT1 and 47 IRF1 motifs (Additional file 1: Table S9).

We studied which of these 80 SNPs affect STAT1/IRF1 binding. First, we utilized the CisGenome “Known Motif Mapping” program to predict which of the variants may modulate STAT1/IRF1 binding motifs (see “Methods” section). CisGenome compares the position weight matrix (PWM) in the JASPAR CORE database and creates likelihood scores for the reference or variant allele. We calculated the fold change in likelihood scores (variant/reference allele) to assess the predicted relative effect. At a cutoff of 1.5-fold, the variant alleles of 34/80 dbSNPs were predicted to modulate the binding affinity of 24 IRF1 motifs and 10 STAT1 motifs (Additional file 1: Table S9).

**Fig. 10:**
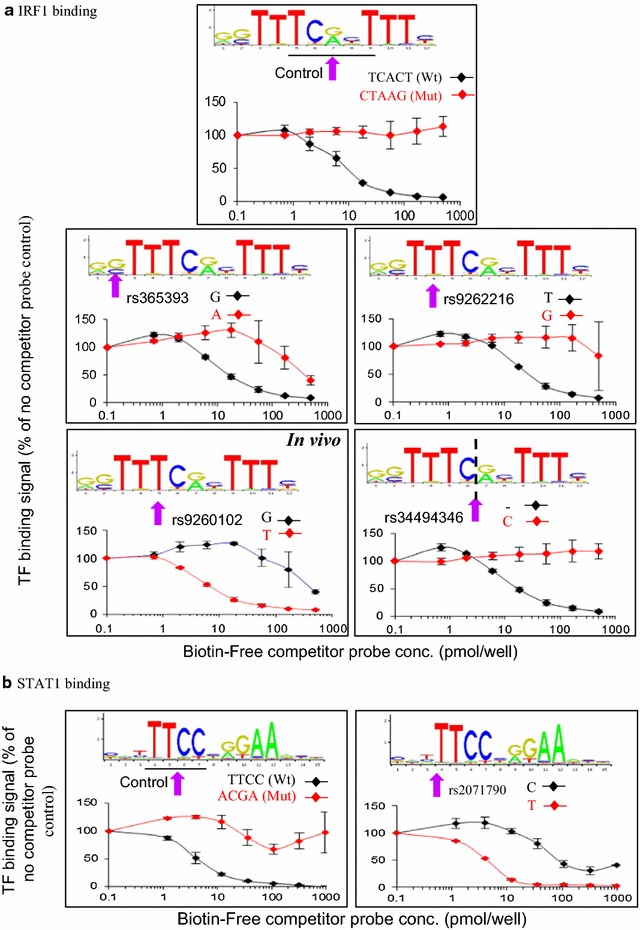
SNPs modulate STAT1 and IRF1 binding in vitro. **a** IRF1, and: **b** STAT1 binding assays. Graphs show STAT1 and IRF1 binding signal to immobilized probes in the presence of different concentrations of competitor probes with either the variant or reference allele. 100% binding is that obtained in the absence of competitor. *Arrows* highlight the affected base (or 4 bases in the control mutated probe). As indicated, rs9260102 was also assessed in vivo (Fig. 11)

To test these predictions in vitro, we developed an ELISA-based DNA affinity assay (see “Methods” section). Canonical STAT1 or IRF1 motif-containing biotinylated 33-mers were immobilized on streptavidin-coated 96-well plates. Cell lysates from HeLa cells exposed to IFNγ for 6 h were mixed with either no or various amounts of Wt (positive control), mutated (negative control), or dbSNP (test) competitor probes, then added to the immobilized biotinylated probe, and the amount of bound TF determined using anti-STAT1 or anti-IRF1 antibody. We tested 4 or 1 SNPs affecting IRF1 or STAT1 sites, respectively (Fig. 10; Additional file 1: Tables S9, S10). Wt IRF1 and STAT1 probes exhibited strong binding with low IC_50_s of 9.6 ± 1.5 or 4.2 ± 0.9 pmol/well, respectively, whereas control mutated probes had minimal/no effect (Fig. 10; Additional file 1: Table S10). 3/6 of the IRF1 SNPs decreased affinity (rs365393, rs9262216, rs34494346) and 1/6 created a strong IRF1 site (rs9260102) (Fig. 10a; Additional file 1: Table S9). The single STAT1 SNP that we tested created a putative binding site, and indeed the T allele of rs2071790 showed high affinity binding (Fig. 10b; Additional file 1: Table S10). Our ChIP-chip data indicated that this SNP lies within an isolated remote IRF1 peak, suggesting that the T allele would convert this regulatory element to a dual STAT1/IRF1 enhancer. In summary, these data show close concordance between the predicted and actual effects of SNPs on STAT1 and IRF1 binding. Thus, it is likely that most of the 34 predicted functional SNPs do in fact alter binding.

### rs9260102 affects IRF1 binding in vivo

**Fig. 11:**
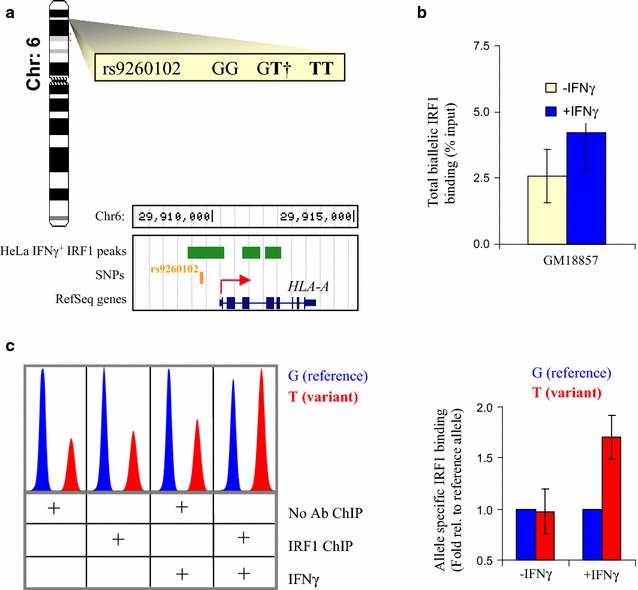
rs9260102 modulates IRF1 binding in vivo. **a** Chromosomal location of rs9260102 and the alleles (strong IRF1 binding in *bold*), and a genome browser view of the SNP, which lies upstream of *HLA*-*A* and within an IFNγ-induced IRF1 Chip-chip peak in HeLa cells. **b** ChIP-qPCR of basal and IFNγ-induced IRF1 recruitment at rs9260102 in GM18857 EBV transformed lymphocytes. **c** Electropherogram on left shows snapshot sequencing of ChIP DNA, with peak quantification plotted on the right (mean ± range, n = 2)

Next we asked if the T allele of rs9260102, which creates an IRF1 site in vitro (Fig. 10a), has this effect in vivo. This SNP lies ~1 kb upstream of the HLA-A locus, within an IFNγ-responsive IRF1 ChIP-chip peak in HeLa cells (Fig. 11a). To test whether it affects IRF1 binding in vivo we employed the EBV-transformed lymphoblastic cell line GM18857, which is heterozygous for rs9260102 (G/T), implying that IRF1 should only bind to one (the T) allele. Treatment with IFNγ for 6 h induced a 1.8-fold increase in the total IRF1 ChIP-qPCR signal (Fig. 11b). Snapshot sequencing revealed that this IFNγ-dependent increase was due solely to elevated binding to the T allele (Fig. 11c). Thus, in silico prediction, an in vitro binding assay, and in vivo allele specific ChIP all show that the G to T switch creates an IRF1 binding site (Fig. 10a; Additional file 1: Table S10).

## Discussion

STAT1 and IRF1 drive the induction of IFN induced genes, but the extent to which they act collectively is unclear. We report that most STAT1 binding (60%) occurs together with IRF1, but most IRF1 binding (72%) is isolated (Fig. 7a). Binding occurs where there are cognate binding motifs (Fig. 7), suggesting that most ChIP signals reflect direct recruitment. Both proximal and remote STAT1 and IRF1 binding is observed at robustly induced ISGs, but not at other loci (Fig. 6). In line with the importance of TF occupancy for responsiveness [46], every responsive locus exhibits a mixture of STAT1 and IRF1 bound enhancers (Fig. 8a). Moreover, dual bound enhancers distinguish induced vs resistant ISGs, whereas single bound enhancers are found with similar frequency at responsive or non-responsive ISGs (Fig. 8). This is not to say, however, that single bound enhancers are irrelevant. For example, while multiple remote *SOCS1* enhancers recruit both TFs, the +50 kb element or promoter are targeted only by STAT1 or IRF1, respectively, yet both are involved in 3D looping (Figs. 4, 5). Similarly, while dual STAT1/IRF1 binding occurs at the active *CIITA* promoter, the −50 kb and +59 kb enhancers recruit only STAT1 or IRF1, respectively, yet contribute to 3D looping and are essential for responsiveness [7]. Indeed, all the responsive genes we surveyed exhibit a mix of STAT1-only, IRF1-only, and STAT1/IRF1 dual enhancers (Fig. 8). Together, these results suggest that IFNγ-responsiveness requires cooperation between enhancers that bind both or either TF, but that STAT1- or IRF1-only enhancers are insufficient for gene induction. Irrespective, it is clear that responsive ISGs integrate information from both STAT1 and IRF1.

Previously, we showed that there is a pre-existing 3D structure at the silent *CIITA* locus, generated through looping between enhancers that subsequently recruit STAT1 and IRF1 upon IFNγ treatment [7]. This was true even in the absence of BRG1, a chromatin remodeling enzyme that is critical to allow stable TF recruitment and thus IFNγ-responsiveness. Subsequent genome-wide analyses indicate that enhancer looping in the poised but silent state is common at inducible loci [47]. We observed the same phenomenon at the IFNγ responsive *SOCS1* locus (Fig. 5). Potentially, these contacts are mediated by pioneer factors that mark responsive enhancers, but their identity at ISGs is unknown. The data here and other studies show that STAT1 and IRF1 can bind some sites in the basal state [21, 28], so in theory, low/unstable binding (undetectable by ChIP) could poise ISG enhancers. It would thus be interesting to perform looping studies at ISGs in STAT1/IRF1 deficient cells. It is of note that the degree to which ISGs were induced in HeLa cells predicted whether they were likely to respond to IFNγ in other cells (Fig. 9). Thus, the chromatin at these genes is accessible in many contexts. The factors that mediate this broad poised, open state may also initiate the basal looping at ISGs.

Over 90% of the disease markers identified in GWAS studies lie within the non-protein-coding regions of the genome [48]. These markers correlate with gene expression [49–52], and lie within gene regulatory regions [53–56]. There is thus considerable interest in identifying SNPs that influence TF binding and, therefore, gene regulation. We identified 80 SNPs within STAT1 or IRF1 motifs, and in silico assessment predicted that 34 may alter binding. In vitro quantification confirmed these predictions in 5/5 cases, arguing that most of/all the other predictions are accurate. The availability of a cell line heterozygous for one such SNP allowed us to test whether the prediction held up in vivo. Indeed, the T allele of rs9260102, which lies just upstream of the HLA-A locus, bound IRF1 whereas the G allele did not, as observed in silico and in vitro. These data serve as proof of principle that in silico prediction is a reliable tool to anticipate the effect of SNPs on STAT1 and IRF1 binding.

## Conclusions

This study provides strong evidence for widespread cooperation between STAT1 and IRF1 at ISGs, and suggests that in silico predictions reliably predict the effect of nucleotide variants on binding in vivo.

## Methods

### Custom oligonucleotide ChIP Tiling array design

A custom oligonucleotide tiling array was designed to cover 11 genomic regions spanning a total of 16 Mb of human genomic DNA in 8 chromosomes (Additional file 1: Table S1). Regions covered from 1 to 5 Mb genomic sequences. Arrays consisted of 50 mers, in quadruplicate, with median probe spacing of 80 bp within non-repetitive DNA regions.

### ChIP on tiled genome arrays (ChIP-chip) and ChIP–quantitative PCR

Details of primers and antibodies used in ChIP assays are in Additional file 1: Tables S11, S12. HeLa-ini1-11 cells (HeLa), were grown as described [12]. Cells were left untreated or exposed to 300 units/ml of human IFN-γ for 6 h (PHC4834, BioSource International, Camarillo, CA, USA). Crosslinked chromatin was sonicated to an average size of about 500 base pairs and was incubated with STAT1 or IRF1 antibody. Bound fragments were purified by ChIP and amplified by ligation-mediated PCR, as described [7], then labeled and hybridized to the arrays. Hybridization intensities were normalized to internal standards and values from quadruplicate spots were averaged. Significantly different intensities between ChIP DNA and input DNA samples in three biological replicates (p < 0.0001) were determined with the Wilcoxon rank-sum test. Peaks representing significantly enriched DNA regions (p < 0.0001) where the ratio of ChIP to input DNA was 1.5-fold or more were visualized with the University of California at Santa Cruz Human (Homo sapiens) Genome Browser (Phast-Cons) and are plotted on a log2 scale. Peaks in a sliding window of 500 base pairs were merged with an in-house Perl script pipeline. ChIP—quantitative PCR was done as described [7], and in all cases, the low background signal obtained with a no-antibody control was subtracted.

### Custom oligonucleotide ChIP tiling array data analysis

Raw intensities from three independent biological replicates, quality assessed by Nimbelgen SignalMap software, were quantile normalized [57], and averaged for each quadruplicate 50 mer. We developed a Wilcoxon Rank Sum test [58] based software to studying the difference between the intensities of the ChIP signal compared to the input DNA signal for each probe within a 500 bp sliding window. Genomic positions with statistically higher intensities (>1.5-fold, p < 10^−4^) from input DNA were merged to form a peak. ChIP-chip data were imported into UCSC genome browser (assembly hg17, NCBI build 35) as two sets of separate tracks for each antibody before and 6 h after IFNγ treatment (http://research.lunenfeld.ca/IFNy).

### STAT1 and IRF1 motif analysis

We mapped STAT1 and IRF1 consensus motifs to the STAT1 and IRF1 ChIP-chip binding regions by using CisGenome “Known Motif Mapping” program. Motif occurrences were determined by using position frequency matrices (PFMs) of STAT1 (ID: MA0137.2) and IRF1 (ID: MA0050.1) from the JASPAR CORE database. The PFMs were converted to pseudo-count matrix for CisGenome’s input. A motif mapping location is selected by the cutoff of a likelihood ratio (LR) > 500. The LR is determined by comparing the motif’s PFM with a background model estimated from input ChIP-chip regions in the 3^rd^ order. Seven sets of ChIP-chip peak regions were mapped and compared with background control regions: STAT1 peaks (92), IRF1 peaks (196), merged STAT1 and/or IRF1 peaks (231), dual STAT1 and IRF1 peaks (56), STAT1 peaks isolated from IRF1 peaks (isolated-STAT1, 56), IRF1 peaks isolated from STAT1 peaks (isolated-IRF1, 140), and basal IRF1 peaks. For each set of peaks, the same number and size of control background regions were randomly sampled from blank regions without any ChIP-chip bindings following the same frequency distribution as the real binding peaks within each cluster segments on the chromosome of ChIP-chip data. The frequencies of regions mapped with motifs were compared between ChIP-chip peaks and random control sites by using the two-sided probability test in R for each paired set of peaks.

### RNA extraction, expression microarray analysis, and Reverse transcriptase-qPCR

RNA extraction and reverse transcription were done from HeLa cells left untreated or at 6, 24 or 48 h after IFNγ treatment as described previously [12]. RNA quality was checked using both Nanodrop (Thermo Fischer Scientific; 260/280 ratio was ≥1.8) and Bioanalyzer (Agilent Inc.; RNA Integrity Number, RIN, ≥ 9.4, range 9.4–9.9). RNA samples were converted to cDNA, followed by a second strand synthesis, and cRNA was prepared using the Ambion kit (Applied Biosystems). The cRNA was column purified and quality was checked using Bioanalyzer (Agilent Inc.). A total of 1.5 µg of cRNA was hybridized to human whole-genome expression arrays (HumanRef-6 Expression BeadChip, Illumina, Inc.) using standard Illumina protocols. Slides were scanned on an Illumina Beadstation and analyzed using BeadStudio (Illumina, Inc). Genes induced by ≥twofold compared to controls and that achieved a differential score of ≥13 were classified as strongly induced ISGs. ISGs which achieved a differential score of ≥13 but fold induction less than 2 were considered weakly induced. Genes reduced by ≥twofold compared to control and achieved a differential score of ≤ −13 were considered strongly reduced. Genes that had a differential score of ≤ −13 but the fold reduction was less than 2 were considered weakly reduced. Three biological replicates were included for each treatment group.

RT-qPCR was performed much as described [59]. Briefly, RNA was extracted from HeLa cells left untreated or at 6, 24 or 48 h after IFNγ treatment using Trizol (Invitrogen), and quality assessed by RIN and OD260/280 as above. cDNA was prepared from 1 mg RNA using random primers and SuperScript RT (Invitrogen). Amplification of cDNA was performed using gene specific primer pairs and SYBER Green Mix (ABI). PCR was ran on Applied Biosystems PRISM 7900HT. Primers were designed in the coding region of each gene (Additional file 1: Table S12). Human genomic DNA was used to prepare calibrators for the quantification of cDNAs. Dissociation curves were inspected to ensure a single product and all PCR products were also tested on a gel to confirm amplification specificity. In addition, no template controls (NTC) were included to ensure the absence of DNA contamination. Gene expression was normalized to multiple house-keeping/reference genes to control for the total amount of RNA. All experiments were done in triplicate.

### Chromosome conformation capture (3C)

The 3C assay was conducted as described [7, 8]. Primer sequences are provided in Additional file 1: Table S12.

### Assessment of TF binding distribution around different classes of ISGs

We compared the distribution of TFBS in the vicinity of es-indISGs and resISGs in STAT1 peaks, IRF1 peaks, and merged STAT1 and IRF1 peaks. We first aligned the TFBS within a range of 400 kb region around the TSS of each gene in a 1 kb resolution, which means that we divided each region into 1 kb windows with the window 0 centered at the TSS and others line up to the 200 kb end upstream and 200 kb end downstream, and then scored the frequency of TFBS at each window as the number of peaks whose center was within the window. If 400 kb extended beyond the ChIP-chip segments, the binding frequencies along the truncated regions were regarded as missing data. Then we plotted the average binding frequencies per 1 kb window versus the relative distance of each to the TSS. Missing values were discarded for averaging the frequencies.

### Defining STAT1 and IRF1 functional SNPs

First, we queried dbSNPs located within the 230 ChIP-chip peaks (UCSC, Build hg19; Track, All SNPs(141); Table, snp141). We defined a total of 6648 dbSNPs. Next we defined SNPs that overlap with STAT1 or IRF1 binding motifs within the 230 ChIP-chip peaks. Then we selected a region of ±50 bp around the SNPs that overlapped with STAT1/IRF1 binding motifs and recovered the DNA sequence of these regions using CisGenome. Then we computationally evaluated the binding affinity of the reference or variant sequence using the likelihood scores obtained from the Cisgenome “known motif mapping” program with the sequences as input to map the STAT1 and IRF1 motif matrix. In some cases the introduction of the variant SNP renders the motif unidentifiable and in this case the sequence of the motif was indicated as “NULL” and the likelihood score was considered as zero (Additional file 1: Table S9). The cutoff value of affinity change was set at 1.5-fold.

### ELISA-based DNA binding affinity assay and ChIP coupled with DNA sequencing

We designed 33-mers with either the reference or variant alleles (Additional file 1: Table S13). Control probes with wild type or dead mutant STAT1 or IRF1 motifs were also included. Probes were ordered biotinylated or biotin-free (competitors). Two pmol of biotinylated probes were immobilized per well of 96-well streptavidin-coated plates. Cell lysates where incubated with different concentrations of the competitor probes (probe-lysate mix) at 4 °C for 3 h to allow STAT1 or IRF1 binding. The probe-lysate mix was then added to streptavidin-coated plates with immobilized biotin probes and incubated overnight at 4 °C. To quantify bound TFs, wells were washed and probed with STAT1 or IRF1 primary antibodies, followed by IR-800 conjugated secondary antibodies. Excess antibodies were washed thoroughly and plates were scanned and quantified using Odyssey Infrared imaging system (LICOR). Signal from no-competitor well is considered as 100% and the % antibody signal was plotted against competitor probe concentration. IC50 values were calculated using Graphpad PRISM 5.2.

For in vivo studies, EBV-transformed lymphoblastic GM18857 cells, cultured as recommended by the supplier (Coriell Biorepositories), were treated with IFNγ for 6 h, fixed and harvested for ChIP analysis. Chromatin was immunoprecipitated using IRF1 antibody and isolated DNA was sequenced using Snapshot sequencing.

## Additional files

**Additional file 1.** Additional tables.

**Additional file 2.** Additional figures.

## Abbreviations

3C: chromosome conformation capture
GAS: IFNγ activation site
IRF1: interferon regulatory factor 1
ISRE: interferon-stimulated response element
HAT: histone acetyltransferase
IFNγ: interferon-γ
ISGs: IFNγ stimulated genes
esISGs: early strong induced ISGs
lsISGs: late strong induced ISGS
ewISGs: early weak induced ISGs
lwISGs: late weak induced ISGs
resISGs: resistant ISGs
potISGs: potential ISGS
IRGs: IFNγ repressed genes
lsIRGs: late strong IRGs
lwIRGs: late weak IRGs
SNPs: single nucleotide polymorphisms
STAT1: signal transducer and activator of transcription 1
TF: transcription factor
TSS: transcription start site
