# Supplementary material for: Properties of STAT1 and IRF1 enhancers and the influence of SNPs
Source: BMC Mol Biol. 2017 Mar 9;18:6. doi: 10.1186/s12867-017-0084-1 (PMC5343312; doi:10.1186/s12867-017-0084-1)
Supplement: Supplementary file 2 — Additional file 2. Additional figures. [file 12867_2017_84_MOESM2_ESM.pptx]

## Slide 1
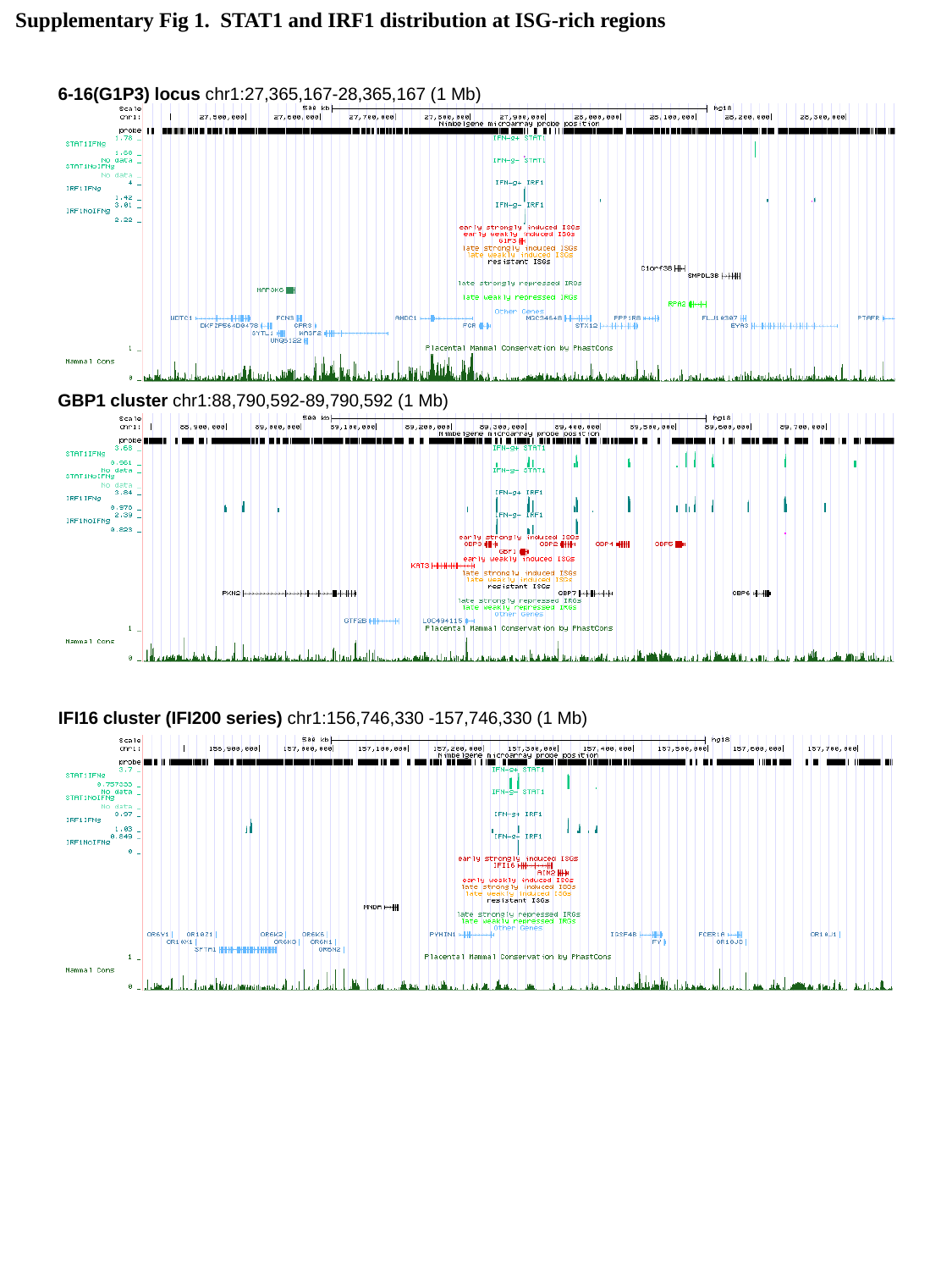

Supplementary Fig 1. STAT1 and IRF1 distribution at ISG-rich regions
6-16(G1P3) locus chr1:27,365,167-28,365,167 (1 Mb)
GBP1 cluster chr1:88,790,592-89,790,592 (1 Mb)
IFI16 cluster (IFI200 series) chr1:156,746,330 -157,746,330 (1 Mb)

## Slide 2
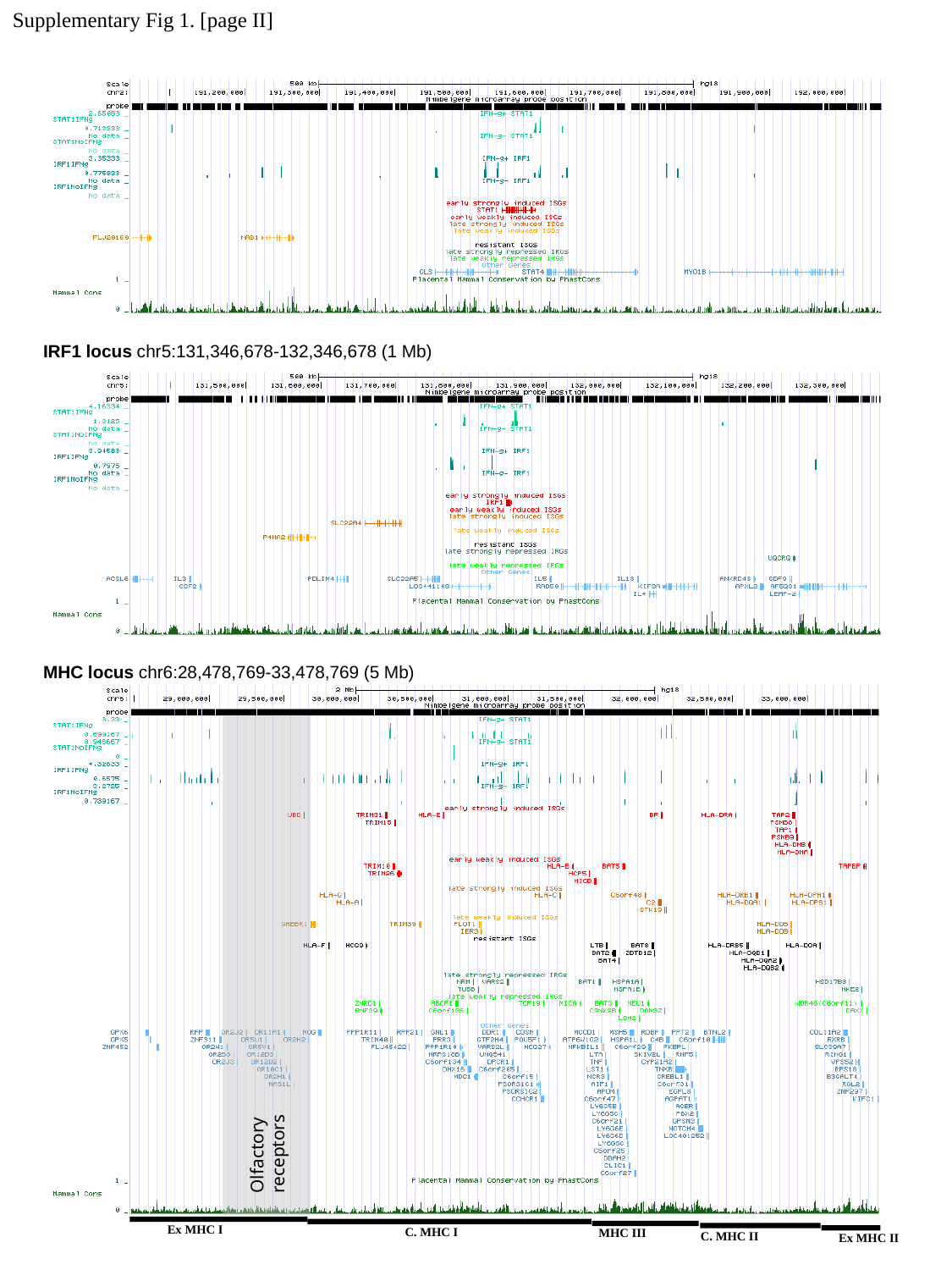

Supplementary Fig 1. [page II]
IRF1 locus chr5:131,346,678-132,346,678 (1 Mb)
MHC locus chr6:28,478,769-33,478,769 (5 Mb)
 Olfactory
 receptors
Ex MHC I
C. MHC I
 MHC III
C. MHC II
Ex MHC II

## Slide 3
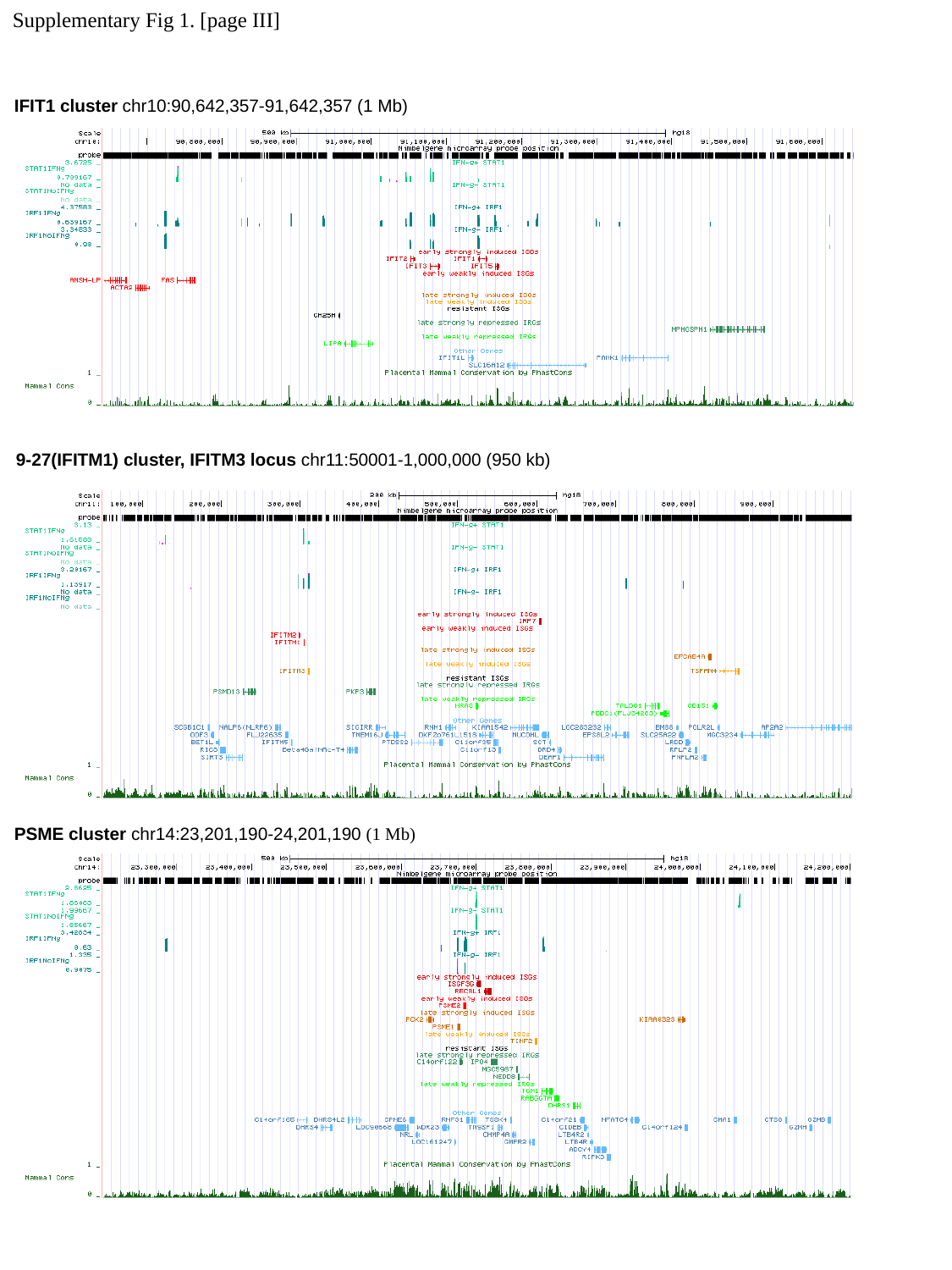

Supplementary Fig 1. [page III]
IFIT1 cluster chr10:90,642,357-91,642,357 (1 Mb)
9-27(IFITM1) cluster, IFITM3 locus chr11:50001-1,000,000 (950 kb)
PSME cluster chr14:23,201,190-24,201,190 (1 Mb)

## Slide 4
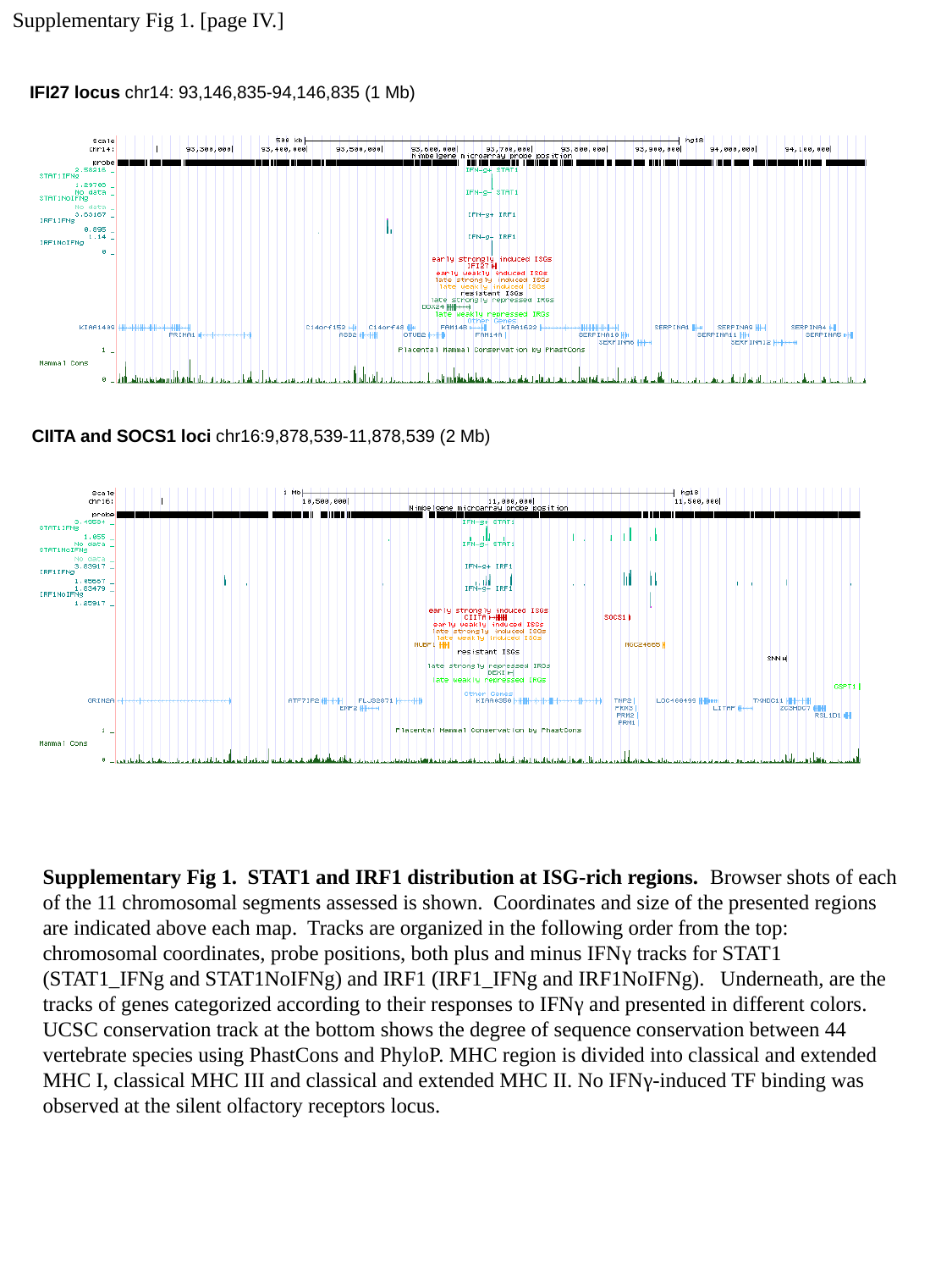

Supplementary Fig 1. [page IV.]
IFI27 locus chr14: 93,146,835-94,146,835 (1 Mb)
CIITA and SOCS1 loci chr16:9,878,539-11,878,539 (2 Mb)
Supplementary Fig 1. STAT1 and IRF1 distribution at ISG-rich regions. Browser shots of each of the 11 chromosomal segments assessed is shown. Coordinates and size of the presented regions are indicated above each map. Tracks are organized in the following order from the top: chromosomal coordinates, probe positions, both plus and minus IFNγ tracks for STAT1 (STAT1_IFNg and STAT1NoIFNg) and IRF1 (IRF1_IFNg and IRF1NoIFNg). Underneath, are the tracks of genes categorized according to their responses to IFNγ and presented in different colors. UCSC conservation track at the bottom shows the degree of sequence conservation between 44 vertebrate species using PhastCons and PhyloP. MHC region is divided into classical and extended MHC I, classical MHC III and classical and extended MHC II. No IFNγ-induced TF binding was observed at the silent olfactory receptors locus.

## Slide 5
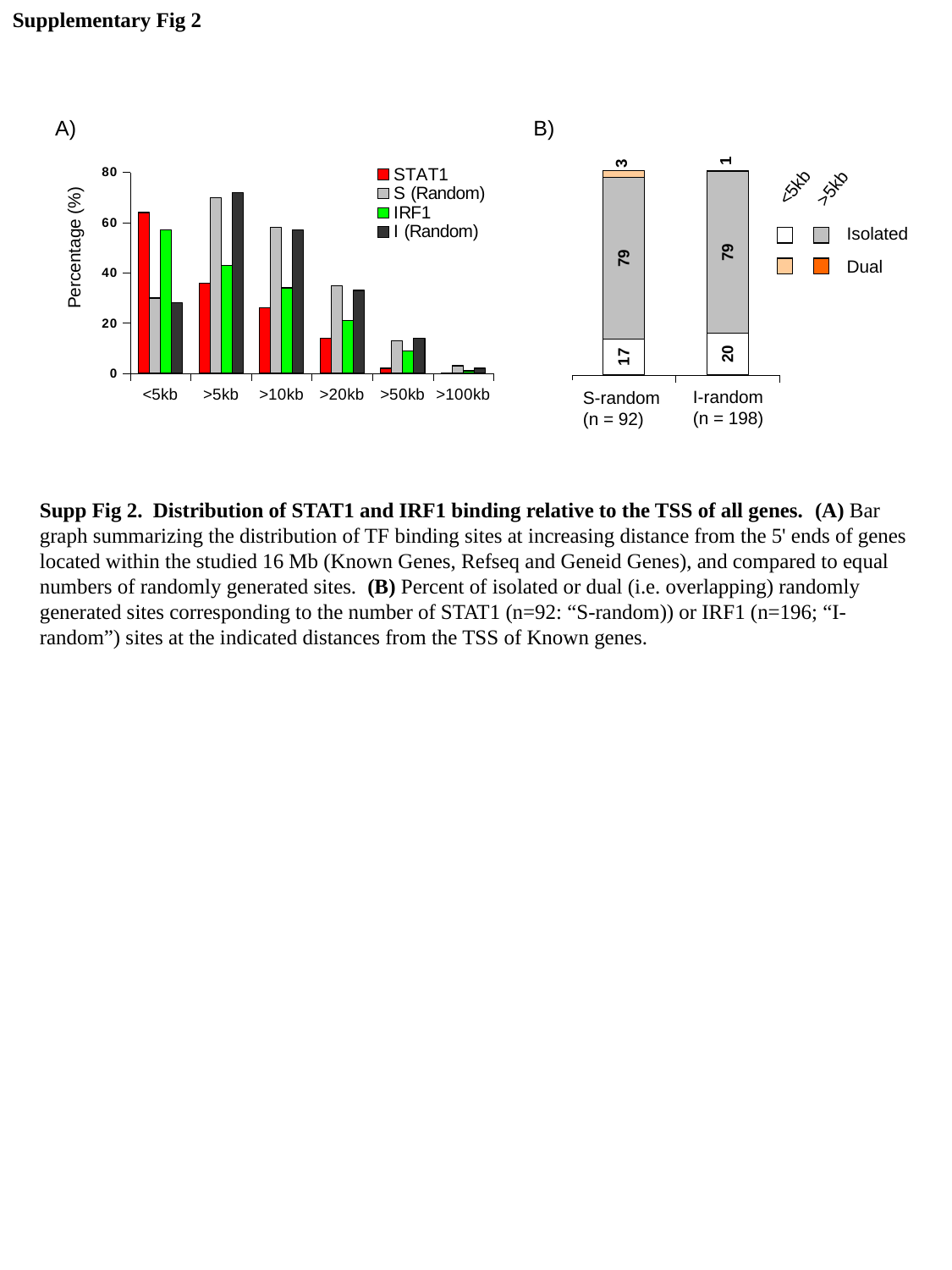

Supplementary Fig 2
A)
B)
### Chart
| Category | Isolated <5kb | Isolated >5kb | Dual <5kb | Dual >5kb |
|---|---|---|---|---|
| STAT1 (n=92) | 17.391304347826086 | 79.34782608695652 | 3.260869565217391 | 0.0 |
| IRF1 (n=196) | 20.408163265306122 | 79.08163265306122 | 0.0 | 0.5102040816326531 |
### Chart
| Category | STAT1 | S (Random) | IRF1 | I (Random) |
|---|---|---|---|---|
| <5kb | 64.0 | 30.0 | 57.0 | 28.0 |
| >5kb | 36.0 | 70.0 | 43.0 | 72.0 |
| >10kb | 26.0 | 58.0 | 34.0 | 57.0 |
| >20kb | 14.0 | 35.0 | 21.0 | 33.0 |
| >50kb | 2.0 | 13.0 | 9.0 | 14.0 |
| >100kb | 0.0 | 3.0 | 1.0 | 2.0 |<5kb
>5kb
Isolated
Dual
Percentage (%)
I-random
(n = 198)
S-random
(n = 92)
Supp Fig 2. Distribution of STAT1 and IRF1 binding relative to the TSS of all genes. (A) Bar graph summarizing the distribution of TF binding sites at increasing distance from the 5' ends of genes located within the studied 16 Mb (Known Genes, Refseq and Geneid Genes), and compared to equal numbers of randomly generated sites. (B) Percent of isolated or dual (i.e. overlapping) randomly generated sites corresponding to the number of STAT1 (n=92: “S-random)) or IRF1 (n=196; “I-random”) sites at the indicated distances from the TSS of Known genes.

## Slide 6
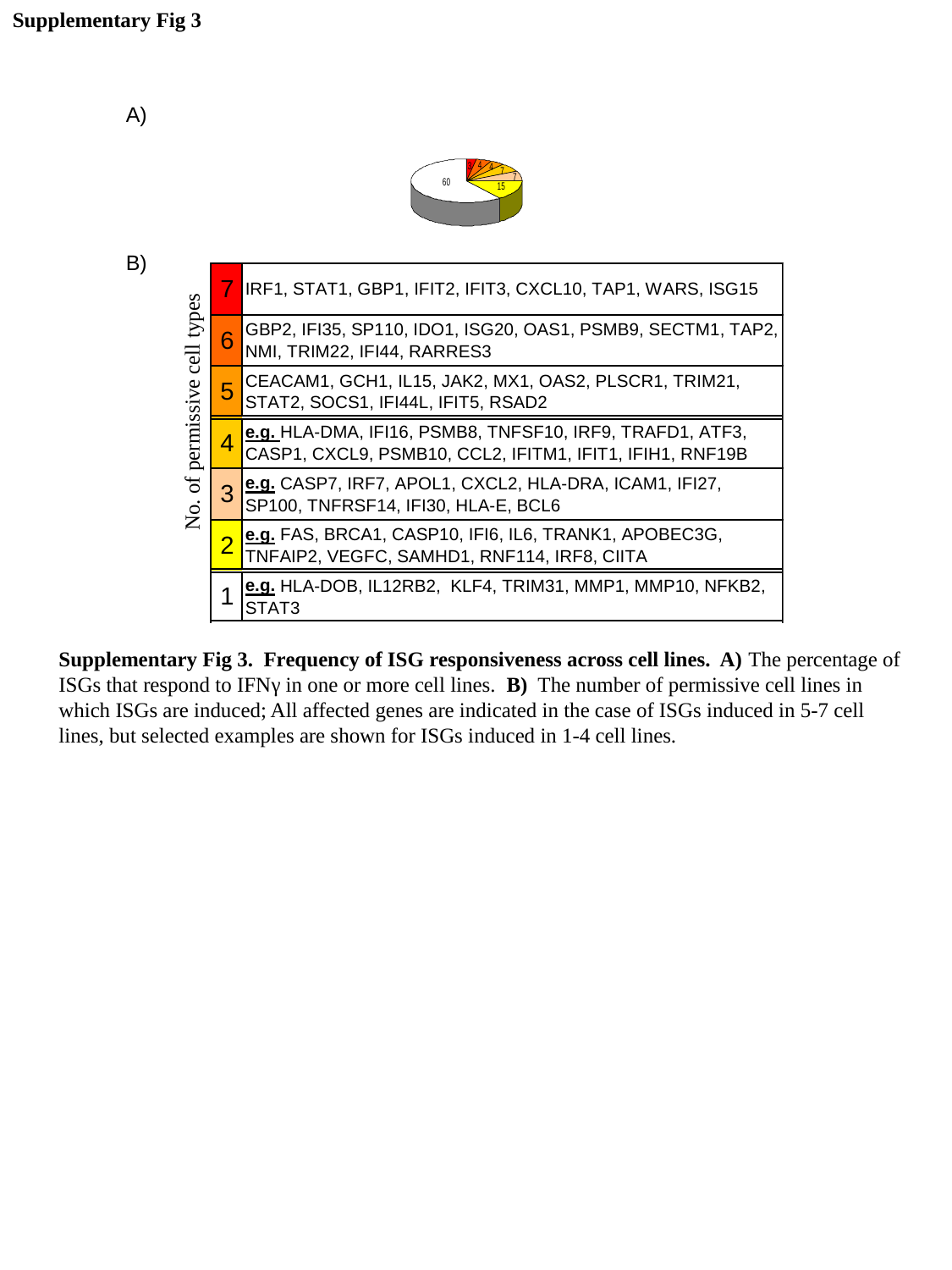

Supplementary Fig 3
A)
No. of permissive cell types
B)
Supplementary Fig 3. Frequency of ISG responsiveness across cell lines. A) The percentage of ISGs that respond to IFNγ in one or more cell lines. B) The number of permissive cell lines in which ISGs are induced; All affected genes are indicated in the case of ISGs induced in 5-7 cell lines, but selected examples are shown for ISGs induced in 1-4 cell lines.
